# Family Leisure, Self-Management, and Satisfaction in Spanish Youth

**DOI:** 10.3389/fpsyg.2019.02231

**Published:** 2019-10-09

**Authors:** Rosa Ana Alonso Ruiz, María Ángeles Valdemoros San Emeterio, Magdalena Sáenz de Jubera Ocón, Eva Sanz Arazuri

**Affiliations:** Department of Education Sciences, University of La Rioja, Logroño, Spain

**Keywords:** leisure, family, self-management, satisfaction, youth

## Abstract

Youth values leisure as a right, a source of growth and integral development, and a context for experimentation. It has been shown that organized leisure leads to more benefits than unorganized leisure; when undertaken (Throughout the document, an attempt will be made to use inclusive language, although “under Law 3/2007 of 22 March, for the effective equality of women and men, any reference to positions, persons or groups included in this document in masculine, are to be understood as including both women and men.”) by the young people themselves, these benefits lead to the development of prosocial skills, self-efficacy, autonomy, and increased independence, personal motivation, and responsibility, as well as acting as a protective factor against risky behaviors. When organized leisure activities are also shared with the family, the benefits influence a positive family environment. This study focused on analyzing the relationship between family-shared leisure practices that are managed by Spanish youth in post-compulsory secondary education and the importance and satisfaction granted to these experiences. The sample consisted of 1,764 post-compulsory secondary education students from all over Spain. Youths responsibility for the organization of their leisure activities, the perception of the relationship between enjoyment of the activity and involvement in its management, the organization of the spaces in which leisure activities are carried out, their satisfaction with them, and the importance attached to shared practice and family experiences were recorded. The results showed a shortage of self-managed youth leisure practices, but increased responsibility, spatial organization, and satisfaction were confirmed when leisure experiences are shared with the family. The need to encourage opportunities for children to self-organize their leisure practices from an early age is commented on.

## Introduction

Leisure is a source of personal and social growth and development (Carrera, 2009; Cuenca, 2009, 2013, 2014; Otero, 2009; Caride, 2012; Clerton de Oliveira et al., 2014; Álvarez et al., 2014) because it is a right that is especially valued by youth, characterized by satisfaction, freedom, and voluntariness (López Ruiz, 2011; Arastegui and Silvestre, 2012; Cuenca and Goytia, 2012). As such, it is carried out in a scenario that is suitable for experimentation (Doistua et al., 2016).

Scientific literature has shown multiple benefits of leisure for the integral development of youths emotional, cognitive, physical, and social aspects (Quintana, 1991; Caride, 1998; Cuenca, 2009; Stebbins, 2012; Freire and Teixeira, 2018; Rodríguez et al., 2018). More specifically, it has also confirmed that organized leisure leads to greater benefits than unorganized leisure (Monteagudo et al., 2017).

Although previous studies (Ortega et al., 2015) indicate that the organization of leisure activities is frequently alien to young people, this research focuses on situations in which leisure experiences are managed by the young people themselves, through their social participation and decision making in the planning and development of these activities. This leads to questioning whether young people have the opportunities for and are interested in the self-management of their leisure time.

In this line, Larson (2000) proposed that most of young peoples time is taken up with academic tasks and periods of structured leisure. Any leisure experience constitutes a fruitful use of leisure time and is considered as a protective factor against risky behaviors and promotes the development of prosocial skills, as well as youth self-efficacy (Casey et al., 2005; García Moya et al., 2012). However, not all practices strengthen youth autonomy and decision-making ability to the same degree. Thus, leisure organized by the young people themselves promotes their independence and requires the assumption of responsibilities in planning, which increases personal motivation and impacts youths life experiences, making them more authentic and allowing them to experiment (Doistua et al., 2016).

However, Rodríguez et al. (2018) noted that young people show little interest in self-managing their leisure practices, and they discovered that youth satisfaction does not increase when they assume responsibilities for the organization of such activities. These results contradict previous studies (Ortega et al., 2015; Doistua and Ried, 2016; Doistua et al., 2016; Lazcano and Caballo, 2016) that found that young people showed greater participation, satisfaction, and degree of commitment in spaces that promoted greater autonomy and opportunities to design and self-manage their leisure experiences rather than in supervised spaces.

Young peoples participation in leisure activities and their assessment of them depend on factors that shape their daily lives (Salazar and Arellano, 2015). In particular, these include aspects such as their schooling, socioeconomic status, or the social and cultural context, as well as certain personal characteristics, such as engagement, responsibility, or commitment, which are related to self-management (Fonseca and Maiztegui-Oñate, 2017; Rodríguez et al., 2018).

There is currently a growing interest in examining the possible relationships between young peoples self-management of leisure and their personal satisfaction and in determining whether such an association is present when leisure activities are shared with the family. In this line are studies on youth satisfaction with the organization of leisure practices with peer groups (Kleiber et al., 2017), but there are very few works focusing on the relationships between youth well-being and the self-organization of leisure when shared with the family, an institution that plays an essential role in young peoples development and well-being (Juang and Silbereisen, 1999; Jackson and Warren, 2000; Demaray and Malecki, 2002; Elzo, 2004; Brazelton and Greenspan, 2005; Álvarez and Rodríguez, 2008; Badenes and López, 2011; Valdemoros et al., 2014).

It is scientifically proven that family-shared leisure leads to multiple benefits, as it provides well-being, improves self-esteem and communicative quality, reduces the chances of engaging in risky behaviors, and promotes encounters, empathy, and generativity among its members (Bell and Bell, 2005; Fiese, 2006; Garmiene et al., 2006; Barnes et al., 2007; Zaborskis et al., 2007; Hebblethwaite and Norris, 2010, 2011; Poff et al., 2010), which are significantly associated with satisfaction with family life (Agate et al., 2009).

On the one hand, family leisure activities have been found to lead to important benefits for family functioning, including the promotion of a positive environment at home, as well as favorable attitudes toward family leisure (Maynard and Harding, 2010; Craig and Mullan, 2012; Grosso et al., 2013; Offer, 2013, 2014; Pinxten and Lievens, 2014; Sanz et al., 2018; Veenstra and Patterson, 2012). On the other hand, a good family atmosphere characterized by warmth and support for young people favors their autonomy in leisure experiences (Ornelas et al., 2007). Lastly, it has been verified that when these activities are organized by the young people themselves, this leads to an increase in self-confidence, social responsibility, and civic sense (Bressler et al., 2005; McCallum et al., 2006; Pinazo and Kaplan, 2007). Therefore, this work is aimed at analyzing the link between young peoples self-management of family-shared leisure activities and the satisfaction and importance they grant to these experiences. The results of this research will facilitate the establishment of lines of action that optimize the benefits derived both from youths self-management and family-shared leisure activities.

## Methodology

### Population and Sample

During the 2013–2014 academic years, a total of 1,055,532 students were enrolled in post-compulsory secondary education in the Spanish state. Given the breadth of the study universe, we decided to select a representative study sample, for which the following parameters were established: sampling error of ±2.3 sigmas, 95% confidence level, and the assumption that *p* = *q* = 0.5. A total of 1,764 subjects made up the final sample, 50.1% female (*n* = 885) and 49.9% male (*n* = 879). Mean age was 17.60± 1.60 years. Of participants, 83% were enrolled in public centers and 17% in private centers. Sixty-seven percent were studying high school, 32.7% middle-grade educational cycles, and 10.3% basic vocational training. Random proportionate cluster sampling was used, taking into account the representativeness of the Spanish state as a whole through the six geographical areas described below:

**A1-Northeast:** composed of Catalonia, Aragon (except for Teruel), and the Balearic Islands.
**A2-Levante:** formed by the Valencian Community, Murcia, and Albacete.
**A3-South:** made up of Andalusia, the Canary Islands, Ceuta, and Melilla.
**A4-Center:** it groups Madrid, Castilla-La Mancha (except for Albacete), Castilla-León (except for León, Palencia, and Burgos), Cáceres, and Teruel.
**A5-Northwest:** it includes Galicia, Asturias, and León.
**A6-North:** it includes Cantabria, Basque Country, La Rioja, Navarre, Burgos, and Palencia.

### Variables

This study is based on six variables. The first five are rated on a 5-point Likert scale the response options: *not at all*, *a little*, *fairly*, *pretty much*, and *very much.*

– Youth responsibility in the organization of their leisure activities: This registers whether the student perceives that he/she participates in the organization of each of his/her leisure activities.
– Perception of the relationship between enjoyment of an activity and engagement in its organization: This registers the students perception of whether greater engagement in the organization of each leisure activity influences the enjoyment of performing them.
– Organization of the spaces in which leisure activities are held: This determines the students degree of responsibility for the preparation of the spaces used for their leisure.
– Satisfaction with each leisure activity practiced: This identifies each youths degree of satisfaction with each of the leisure activities indicated.
– Importance of the activity: This shows the degree of importance of each leisure activity for each student.
– Practiced in the family: This dichotomous variable, composed of the categories *yes* and *no*, identifies which activities are shared with a family.

### Instrument

For data collection, we created an *ad hoc* questionnaire. This instrument was validated through a pilot test with students from eight autonomous communities and through experts judgment, involving 14 researchers of leisure from seven Spanish universities.

### Procedure

Each of the General Directors of Education of the participating Autonomous Communities was informed through a letter about the purposes of the study. After the administrative authorities had granted permission, two researchers trained to ensure the standardized application traveled to each of the randomly selected schools to apply the instruments.

### Data Analysis

On a first level, a descriptive analysis was carried out to calculate the frequencies, means, and standard deviations in order to describe the students responsibility and autonomy in the organization of their leisure, as well as the satisfaction and importance they attach to these activities, and the amount of young people who share leisure with their family. The students were classified as sharing leisure with their family (FL) or not sharing their leisure with their family (NFL). After this descriptive analysis, we performed a second bivariate inferential level to determine possible significant differences in responsibility and autonomy in the organization of leisure activities and the satisfaction and importance granted to them as a function of whether or not they are practiced with family members. This was performed with Students *T*-test for independent samples. Finally, through correlational analysis, we determined a possible linear relationship between students responsibility for the management of family-shared leisure activities and the satisfaction they feel and the importance they attach to these activities. The level of significance established for this study was *p* < 0.05.

## Results

Post-compulsory high school students considered that they assume little responsibility for the organization of their leisure activities (*M* = 2.26 ± 1.61) and they did not believe that if they engaged more actively in this issue, they would enjoy the activities more (*M* = 1.40 ± 1.24). In general, they did not carry out their leisure in self-organized spaces (*M* = 1.85 ± 1.52), they were somewhat satisfied with their main activities (*M* = 2.94 ± 1.73), but they did not consider them to be very important (*M* = 2.55 ± 1.59).

On the other hand, 46.5% of the students in post-compulsory secondary education reported sharing some leisure activity with their families. In this sense, 31.1% practiced one leisure activity with their family, compared with 11.2% who indicated two family leisure activities and 4.3% who claimed they practiced more than three activities with their family.

Bivariate relational analysis showed that post-compulsory high school students take on more autonomy for organizing their leisure activities when they are shared with the family (*M*_F__L_ = 2.67 ± 0.910 vs. *M*_NFL_ = 1.90 ± 1.297) (Table 1).

**TABLE 1:** Summary of Students *T*-test for independent samples: autonomy for organizing leisure activities based on whether or not the activity is shared with the family.

	**Levenes test for variance equality**		***T*-test for equal means**			***M* ± *SD***
	***F***	***p*^1^**	***T***	***df***	***p***	
I am responsible for organizing the activity	213.88	0.125	−10.09	1,455.89	0.000	Family leisure (FL) = 2.67 ± 0.910
						Non-family leisure (NFL) = 1.90 ± 1.297

*^1^Levenes test: *p* > 0.05 in all cases, so the equality of variances is assumed.*

In the same vein, when these young people practice family leisure activity, they are more likely to consider that their autonomy in the organization of the activity provides more enjoyment (*M*_FL_ = 1.68 ± 1.41 vs. *M*_NFL_ = 1.16 ± 1.00) (Table 2).

**TABLE 2:** Summary of Students *T*-test for independent samples: enjoyment of the activity in relation to participation in its organization as a function of whether or not the activity is shared with the family.

	**Levenes test for variance equality**		***T*-test for equal means**			***M* ± *SD***
	***F***	***p*^1^**	***T***	***df***	***p***	
I would enjoy the activity more if I engaged more actively in its organization	125.788	0.284	−8.896	1,762	0.000	Family leisure (FL) = 1.68 ± 1.41 Non-family leisure (NFL) = 1.16 ± 1.00

*^1^Levenes test: *p* > 0.05 in all cases, so the equality of the variances is assumed.*

Family-shared leisure is practiced significantly more in spaces organized by post-compulsory high school students than those activities that are not shared with the family members (*M*_*FL*_ = 2.25 ± 1.74 vs. *M*_NFL_ = 1.49 ± 1.20) (Table 3).

**TABLE 3:** Summary of Students *T*-test for independent samples: development of leisure in spaces organized by youth as a function of whether or not the activity is shared with the family.

	**Levenes test for variance equality**		***T*-test for equal means**			***M* ± *SD***
	***F***	***p*^1^**	***T***	***df***	***p***	
I practice this activity in spaces that I organize myself	243.298	0.468	−10.69	1,762	0.000	Family leisure (FL) = 2.25 ± 1.74 Non-family leisure (NFL) = 1.49 ± 1.20

*^1^Levenes test: *p* > 0.05 in all cases, so the equality of the variances is assumed.*

There is a significant difference in the degree of satisfaction with leisure activities depending on whether or not they are shared with family members. Students are more satisfied with family-shared leisure activities (*M*_FL_ = 1.68 ± 1.41 vs. *M*_NFL_ = 1.16 ± 1.00) (Table 4).

**TABLE 4:** Summary of Students *T*-test for independent samples: satisfaction with leisure activity depending on whether or not it is shared with the family.

	**Levenes test for variance equality**		**T-test for equal means**			***M* ± *SD***
	***F***	***p*^1^**	***T***	***df***	***p***	
I would enjoy the activity more if I engaged more actively in its organization	61.777	0.169	−12.36	1,762	0.000	Family leisure (FL) = 3.47 ± 1.85 Non-family leisure (NFL) = 2.49 ± 1.46

*^1^Levenes test: *p* > 0.05 in all cases, so the equality of the variances is assumed.*

Post-compulsory high school students perceived that their family activities are significantly more important to them than those leisure experiences that they do not share with family members (*M*_FL_ = 3.00 ± 1.77 vs. *M*_NFL_ = 2.16 ± 1.30) (Table 5).

**TABLE 5:** Summary of Students *T*-test for independent samples: importance of activity for the student depending on whether or not it is shared with the family.

	**Levenes test for variance equality**		***T*-test for equal means**			***M* ± *SD***
	***F***	***p*^1^**	***T***	***Df***	***p***	
I would enjoy the activity more if I engaged more actively in its organization	87.408	0.352	−11.57	1,762	0.000	Family leisure (FL) = 3.00 ± 1.77 Non-family leisure (NFL) = 2.16 ± 1.30

*^1^Levenes test: *p* > 0.05 in all cases, so the equality of the variances is assumed.*

**FIGURE 1:**
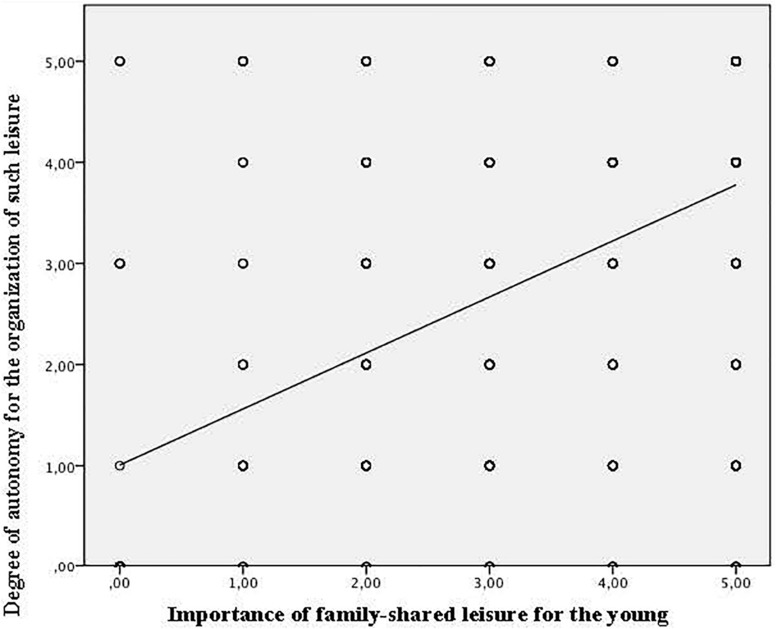
Degree of dispersion: Satisfaction with family-shared leisure and degree of autonomy for the organization of such leisure.

The Pearson correlation analysis yielded positive linear relationships between satisfaction with family-shared leisure activities and autonomy for the organization of the activity (*r* = 0.631, *p* = 0.000), participation in the organization of spaces (*r* = 0.538, *p* = 0.000), the belief that participating more in the organization of the activity would provide greater enjoyment (*r* = 0.407, *p* = 0.000), and the importance that this family activity has in their lives (*r* = 0.707, *p* = 0.000) (Figure 1).

**FIGURE 2:**
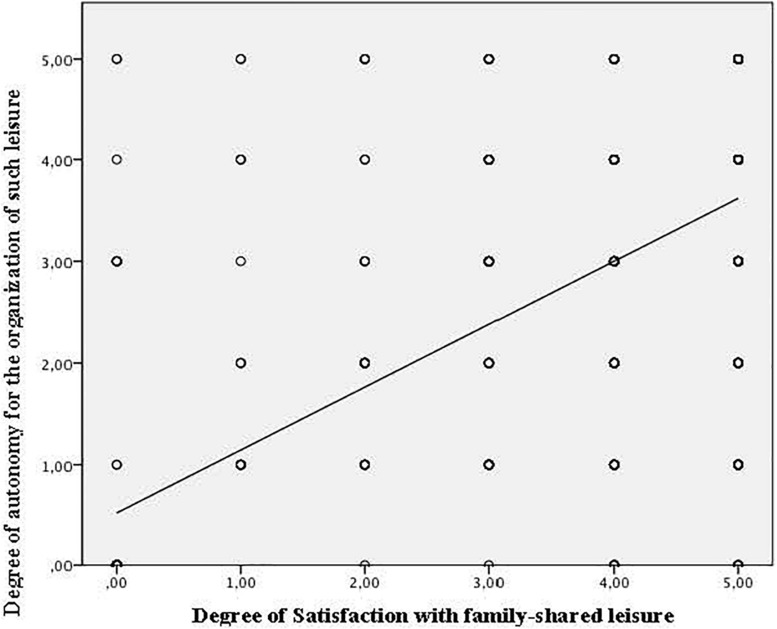
Degree of dispersion: Importance of family-shared leisure for the young people and degree of autonomy for the organization of such leisure.

The Pearson correlation analysis also yielded positive linear relationships between the importance of this family activity for the young people and the autonomy for organizing the activity (*r* = 0.538, *p* = 0.000), responsibility in the organization of the spaces (*r* = 0.474, *p* = 0.000), and the belief that participating more actively in the organization of the activity would provide greater enjoyment (*r* = 0.415, *p* = 0.000) (Figure 2).

## Discussion

The findings of this study verify the low levels of self-management in young peoples leisure; that is, there are few activities in which youth engages socially in the participation and decision making of the planning and the development of the activities. This is in line with the work of Rodríguez et al. (2018), who corroborated the scarce interest shown by youth in the organization of leisure activities.

Although previous studies (Doistua et al., 2016) ensure that self-managed leisure activities provide greater motivation and leads to more authentic experiences that facilitate experimentation, our research found that young people perceive that taking on more autonomy for the organization of their leisure does not increase their satisfaction. This is in line with the results of the study of Rodríguez et al. (2018), who also stated that the degree of satisfaction does not increase when students assume the responsibility for organizing and managing their leisure practices.

Regarding the family sphere, this study shows that young people do share leisure activities with their families, which is consistent with previous research (Ponce de León et al., 2015; Sanz et al., 2018) that verifies the desire to practice this type of leisure, but it disagrees with other authors (Berntsson and Ringsberg, 2014) who state that few young people acknowledge that they share their leisure experiences with direct relatives. This reveals a lack of agreement in the scientific literature on this issue.

Despite finding low levels in the self-organization of youth leisure, leading to lower commitment to its development and to poor management of the spaces in which leisure activities are practiced, our results nonetheless support an increase in the rating of responsibility and spatial organization when these leisure experiences are shared with the family. We also highlight the presence of a positive relationship between young peoples self-management of leisure and their personal satisfaction when these activities are practiced in the family. To this conclusion is added the fact that these family-shared leisure experiences are felt to be more important than those that are not shared. Although previous studies (Kleiber et al., 2017) show that leisure activities organized and practiced with the peer group achieve higher levels of well-being in young people, there are no conclusive results linking personal satisfaction to family-shared leisure activity. This is a limitation of this investigation, and we recommend continuing this line of study in the future.

Finally, it is confirmed that the family is a privileged area for the construction of the children’s leisure (Valdemoros et al., 2014) and that family-shared leisure provides benefits for family functioning (Agate et al., 2009; Maynard and Harding, 2010; Craig and Mullan, 2012; Veenstra and Patterson, 2012; Grosso et al., 2013; Offer, 2013, 2014; Pinxten and Lievens, 2014; Sanz et al., 2018). The numerous benefits derived from leisure experiences when self-managed by young people are also shown (Ortega et al., 2015; Doistua and Ried, 2016; Doistua et al., 2016; Lazcano and Caballo, 2016). This research has revealed an increase in well-being when such practices take place within the family nucleus and the school, and the youngsters assume responsibility for their management and organization. This justifies the need to promote from an early age actions that offer opportunities to plan and develop family leisure experiences, involving the youngest children in simple decisions about the form of the activity and the preparation of the material required to practice it, and to consider infancy as a favorable period to lay the groundwork for the acquisition of behaviors and competences to participate in leisure practices.

## Data Availability Statement

The datasets generated for this study are available on request to the corresponding author.

## Ethics Statement

The studies involving human participants were reviewed and approved by Comité de Ética de la Universidad de La Rioja. Written informed consent to participate in this study was provided by the participants legal guardian/next of kin.

## Author Contributions

All authors have contributed equally to each and every part of this manuscript, equally involved in the bibliographic search and review, as well as in the reflective debate and drafting of the theoretical bases, involved in the methodological design and statistical data analysis, in charge of data collection in the different Spanish autonomous communities, and participated in the interpretation and drafting of the results, as well as in the discussion and conclusions of the study carried out, through their shared dialogue.

## Conflict of Interest

The authors declare that the research was conducted in the absence of any commercial or financial relationships that could be construed as a potential conflict of interest.
